# Innate immune response against vector-borne bunyavirus infection and viral countermeasures

**DOI:** 10.3389/fcimb.2024.1365221

**Published:** 2024-04-22

**Authors:** Minghua Li

**Affiliations:** ^1^ Department of Pathology, University of Texas Medical Branch, Galveston, TX, United States; ^2^ Center for Biodefense and Emerging Infectious Diseases, University of Texas Medical Branch, Galveston, TX, United States; ^3^ Center for Tropical Diseases, University of Texas Medical Branch, Galveston, TX, United States; ^4^ Institute for Human Infections and Immunity, University of Texas Medical Branch, Galveston, TX, United States

**Keywords:** bunyaviruses, innate immunity, interferon, interferon-stimulated gene, antagonism

## Abstract

Bunyaviruses are a large group of important viral pathogens that cause significant diseases in humans and animals worldwide. Bunyaviruses are enveloped, single-stranded, negative-sense RNA viruses that infect a wide range of hosts. Upon entry into host cells, the components of viruses are recognized by host innate immune system, leading to the activation of downstream signaling cascades to induce interferons (IFNs) and other proinflammatory cytokines. IFNs bind to their receptors and upregulate the expression of hundreds of interferon-stimulated genes (ISGs). Many ISGs have antiviral activities and confer an antiviral state to host cells. For efficient replication and spread, viruses have evolved different strategies to antagonize IFN-mediated restriction. Here, we discuss recent advances in our understanding of the interactions between bunyaviruses and host innate immune response.

## Introduction

The *Bunyavirales* order contains a large group of medically relevant, emerging and re-emerging RNA viruses known as bunyaviruses. The order includes more than 500 viruses that are transmitted by arthropod vectors including mosquitoes, sandflies and ticks or rodents and infect a variety of mammals, insects and plants (Elliott, 2014; Abudurexiti et al., 2019). As of December 2023, the *Bunyavirales* order comprises 14 families (https://ictv.global/taxonomy), of which 5 families (*Arenaviridae*, *Hantaviridae*, *Nairoviridae*, *Peribunyaviridae*, and *Phenuiviridae*) contain viral pathogens that can cause significant diseases in human, such as fever, hemorrhagic disease, encephalitis, and severe respiratory disease (Elliott, 1997). Some of these viruses include Hantaan virus (HTNV) and Andes virus (ANDV) in the *Hantaviridae* family; Crimean-Congo hemorrhagic fever virus (CCHFV) in the *Nairoviridae* family; Bunyamwera virus (BUNV), La Crosse virus (LACV) and Oropouche virus (OROV) in the *Peribunyaviridae* family; Guertu virus (GTV), Heartland virus (HRTV), severe fever with thrombocytopenia syndrome virus (SFTSV) and Rift Valley fever virus (RVFV) in the *Phenuiviridae* family. Several members in the *Arenaviridae* family are highly pathogenic viruses that are transmitted by rodents. In this review, we mainly focus on vector-borne bunyaviruses.

Bunyavirus replication occurs in the cytoplasm, making these viruses sensitive to several cytosolic innate immune sensors. Upon binding to viral ligands, these sensors initiate downstream interferon (IFN) signaling pathways to block viral infection. In response, viruses have evolved a variety of strategies to antagonize host innate immunity and facilitate their spread in the host cells. Here, we review how host innate immune system detects and restricts bunyavirus infection and discuss the mechanisms by which bunyaviruses counteract host IFN response.

## The genome organization and life cycle of bunyavirus

The genome of bunyavirus consists of three single-stranded, negative-sense RNA segments: large (L), medium (M) and small (S) segment. All bunyaviruses encode four structural proteins: the RNA-dependent RNA polymerase (L) on L segment, the envelope glycoproteins (Gn and Gc) on M segment and the nucleocapsid protein (N) on S segment. In addition, some bunyaviruses can encode several nonstructural proteins, including NSm on M segment and NSs on S segment (Hulswit et al., 2021). The structural proteins of bunyavirus are required for viral entry, genome replication and assembly while nonstructural protein NSs is involved in the antagonism of host immune response (Elliott and Weber, 2009).

The infection cycle of bunyavirus begins with the binding of viral envelope glycoproteins Gn and Gc to cellular receptors on the host cell surface. The receptor engagement triggers the internalization of virus into endosomes via endocytosis in a clathrin-dependent or clathrin-independent manner. The acidic environment of the endosomes induces the conformational changes of viral glycoproteins, leading to the endosomal fusion. The membrane fusion is mediated by the viral envelope glycoprotein Gc. Gc is a class II membrane fusion protein that harbors three domains (I, II and III). These domains form fusion loops to insert into cellular membrane and drive virus-cell fusion (Hellert et al., 2023). The fusion of viral and cell membranes forms a fusion pore to allow the release of viral RNA into the cytoplasm of host cells. The viral negative-sense genomic RNA is first transcribed into mRNAs by viral L protein. These viral mRNAs are subsequently translated into viral proteins using host cell translation machinery. Once viral proteins are synthesized, viral RNA replication begins. Viral genomic RNA serves as a template to synthesize the complementary positive-sense antigenomic RNA, which is then used to generate progeny negative-sense genomic RNA. The newly synthesized viral RNA and viral proteins are assembled at the Golgi apparatus. Subsequently, the progeny virions are transported by the secretory vesicles to the cells surface, where they are released into the extracellular space via exocytosis (Walter and Barr, 2011; Boshra, 2022).

## Induction of IFN by bunyavirus infection

Upon viral infection, the host innate immune response is initiated by recognition of virus-associated molecules, known as pathogen-associated molecular patterns (PAMPs), via host-encoded pattern recognition receptors (PRRs) (Kawai and Akira, 2006; Iwasaki, 2012). Those viral components including genomic DNA or RNA and double-stranded RNA (dsRNA) intermediates produced during viral replication can be detected by diverse PRRs depending on their subcellular location. In the cytoplasm, retinoic acid-inducible gene I (RIG-I)-like receptors (RLRs) are the major PRRs that sense RNA virus infection (Chan and Gack, 2016). RLRs are composed of three members: RIG-I, melanoma differentiation-associated protein 5 (MDA5) and laboratory of genetics and physiology 2 (LGP2). All RLRs have a central helicase domain and a carboxy-terminal domain (CTD), both of which are required for RNA binding. In addition, RIG-I and MDA5 contain two amino-terminal caspase activation and recruitment domains (CARDs) that are involved in signal transduction. RIG-I has been shown to sense short dsRNA that contains triphosphate (PPP) and uncapped diphosphate (PP) groups at the 5’ end of viral RNA while MDA5 detects long dsRNA structures. LGP2 that lacks a CARD domain was found to regulate the sensing activities of RIG-I and MDA5 (Liu and Gack, 2020; Rehwinkel and Gack, 2020). Upon binding to their RNA ligands, RIG-I and MDA5 interact with adaptor protein mitochondrial antiviral-signaling protein (MAVS). MAVS triggers the activation of TANK-binding kinase 1 (TBK1) or IκB kinase complex, leading to the phosphorylation of IFN regulatory factor 3 (IRF3) and IRF7, and nuclear factor-κB (NF-κB), respectively. The activated IRF3, IRF7 and NF-κB then translocate into nucleus and induce the transcription of type I and type III IFNs as well as other pro-inflammatory cytokines and chemokines (Thompson et al., 2011; Jensen and Thomsen Allan, 2012).

RLRs play a critical role in immune recognition of bunyavirus infection. RIG-I was shown to be the major PRR that sensed the infection of LACV, RVFV and SFTSV to induce innate immune response, as evidenced by decreased IFNs and ISGs induction in RIG-I-depleted cells (Habjan et al., 2008; Verbruggen et al., 2011; Weber et al., 2013; Min et al., 2020). Enzymatic degradation of viral 5’-PPP RNA abolished RIG-I-mediated IFN induction, suggesting that viral 5’-PPP RNA is the ligand of RIG-I activation during these virus infection (Habjan et al., 2008; Min et al., 2020). Furthermore, RIG-I was found to directly bind to N proteins of LACV and RVFV and recognize N-associated viral 5’-PPP RNA to activate early IFN response (Weber et al., 2013). Although viral 5’-PPP RNA has been considered as the major PAMP that activates RIG-I signaling, some bunyaviruses that do not have 5’-PPP in their genomic RNA still triggered RIG-I-dependent IFN response, suggesting that these viruses may contain additional PAMPs that function as RIG-I ligands (Lee et al., 2011). Indeed, the dsRNA-like secondary structures of HTNV N RNA was shown to activate RIG-I during viral replication (Lee et al., 2011; Spengler et al., 2015). HTNV also induced the expression of long noncoding RNA NEAT1 to promote RIG-I signaling in response to viral infection (Ma et al., 2017). Interestingly, CCHFV that harbors 5’-monophosphate RNA was able to stimulate IFN signaling in a RIG-I dependent manner (Spengler et al., 2015). However, the role of these putative PAMPs in RIG-I-mediated immune response needs to be further investigated. In addition to RIG-I, other PRRs including MDA5 and Toll-like receptor 3 (TLR3) have been shown to be involved in the immune sensing of SFTSV and HTNV, respectively (Handke et al., 2009; Min et al., 2020).

Although cytosolic DNA sensor cyclic GMP-AMP synthase (cGAS) and its adaptor protein stimulator of interferon genes (STING) are considered as key immune molecules to sense DNA viruses, recent studies suggest that STING plays a role in the infection of RNA viruses, including bunyaviruses. STING was found to be involved in nuclear scaffold attachment factor A (SAFA)-mediated immune response against SFTSV infection (Liu et al., 2021). Furthermore, STING was activated during HTNV infection, triggering autophagy to block viral infection. Interestingly, RIG-I but not cGAS was required for HTNV-induced STING activation, suggesting a crosstalk between RNA and DNA sensing pathways to detect bunyavirus infection (Wang et al., 2023).

## Restriction of bunyavirus infection by interferon-stimulated genes

**Figure 1:**
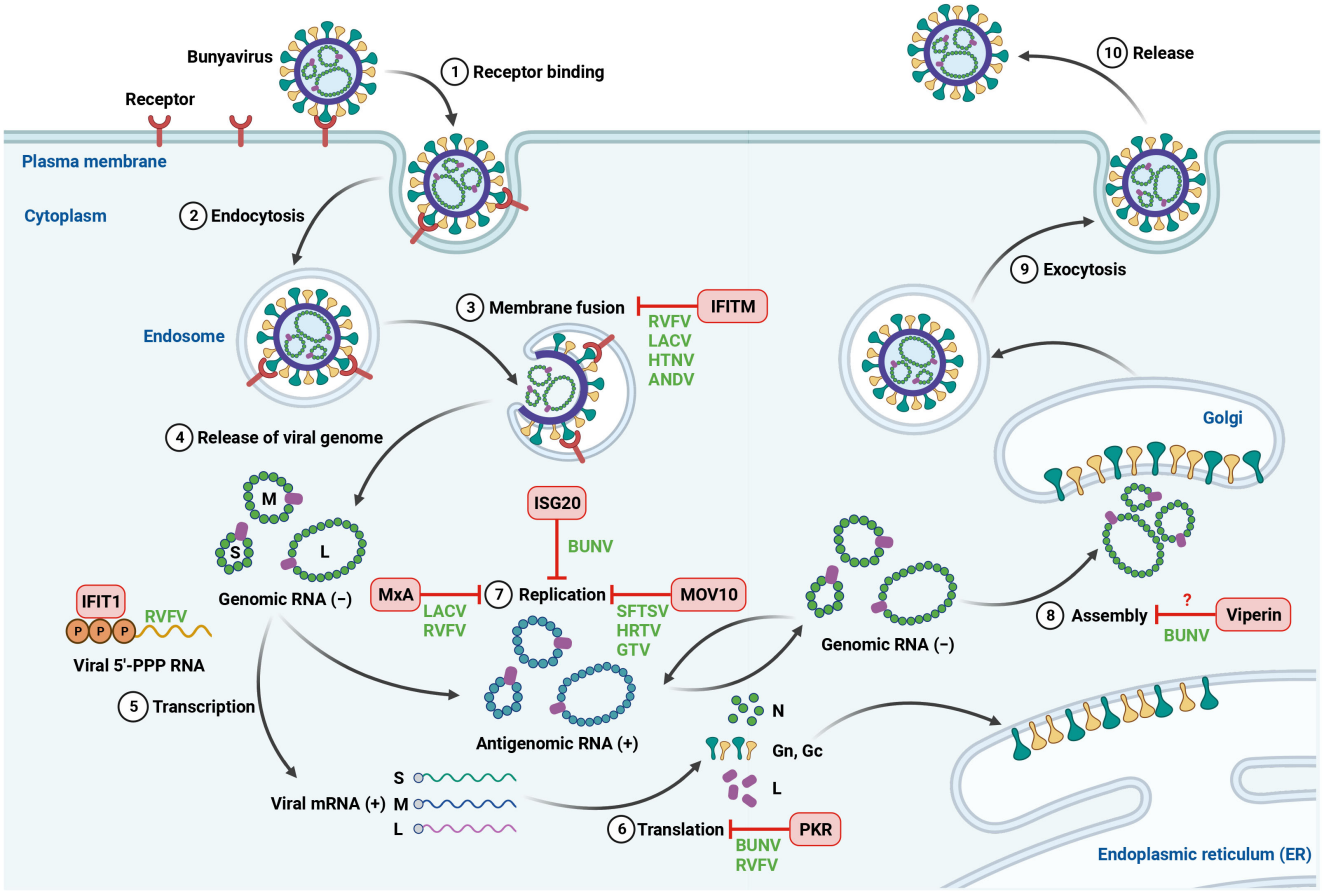
Inhibition of bunyavirus infection by host antiviral ISGs. The life cycle of bunyavirus is initiated by the binding of viral glycoproteins to cellular receptors on the cell surface. The virus is then internalized by endocytosis, followed by trafficking within endosomes, where the endosomal fusion occurs. The fusion between viral and cell membranes allows the release of viral genome into the cytoplasm. The negative-sense genomic RNA is first transcribed into mRNA, which is used as a template for viral protein synthesis. Viral genomic RNA then functions as a template to synthesize positive-sense antigenomic RNA for genome replication. The newly synthesized viral genomic RNA and viral proteins are assembled and processed at the Golgi. The mature viral particles are released from the infected cells via exocytosis. Host-encoded ISGs can block bunyavirus infection at different steps of viral life cycle. Created with BioRender.com.

The induction of ISGs is one of the major responses to IFN signaling. Upon production, type I and type III IFNs bind to their receptors IFNAR1/IFNAR2 or IFNLR1/IL-10R2, respectively, and trigger the phosphorylation of Janus kinase 1 (JAK1) and tyrosine kinase 2 (TYK2), leading to the phosphorylation of signal transducers and activators of transcription 1 and 2 (STAT1 and STAT2). The phosphorylated STAT1 and STAT2 recruit IRF9 to form the IFN-stimulated gene factor 3 (ISGF3). The ISGF3 subsequently translocates to the nucleus and binds to IFN-stimulated response elements (ISRE) in the upstream promoter regions of ISGs, resulting in the induction of more than 300 ISGs (Schneider et al., 2014; Schoggins, 2019). Many of these ISGs have antiviral activities and can block the infection of a wide range of viruses. Indeed, a number of ISGs were found to suppress bunyavirus infection at different stages of viral life cycle, including viral entry, translation and replication (**Figure 1**). Not surprisingly, bunyaviruses can directly target antiviral ISGs to overcome their restriction. Here, we describe several ISGs that play an antiviral role during bunyavirus infection.

IFN-induced protein with tetratricopeptide repeats 1 (IFIT1): The human IFIT family is comprised of 4 members (IFIT1, IFIT2, IFIT3 and IFIT5) and has been shown to play a role during viral infection (Terenzi et al., 2008; Fensterl and Sen, 2010; Pichlmair et al., 2011; Kimura et al., 2013). Using affinity purification followed by mass spectrometry, Pichlmair et al. identified IFIT proteins as binding partners of 5′-PPP RNA, a viral PAMP that can be recognized by host immune sensors to initiate IFN signaling pathways (Pichlmair et al., 2011). The authors found that IFIT1 and IFIT5 directly bound to 5′-PPP RNA while IFIT2 and IFIT3 indirectly associated with 5′-PPP RNA via an IFN-dependent complex consisted of IFIT1, IFIT2 and IFIT3. This provided the first hint that IFIT proteins may be involved in immune response against viruses generating 5′-PPP RNA. Indeed, loss of IFIT proteins promoted the infection of several viruses containing 5′-PPP RNA, including RVFV, vesicular stomatitis virus (VSV) and influenza A virus (IAV) but had no effect on encephalomyocarditis virus (EMCV) that lacks 5′-PPP RNA in its life cycle (Pichlmair et al., 2011). These data suggest that IFIT proteins function as restriction factors against viruses harboring 5′-PPP RNA. However, the precise molecular mechanism of how IFIT proteins target 5′-PPP RNA to block viral infection is not fully understood.

IFN-induced transmembrane proteins (IFITMs): To date, five members of human IFITM family have been characterized: IFITM1, IFITM2, IFITM3, IFITM5 and IFITM10. Three of them (IFITM1, IFITM2, IFITM3) are IFN inducible and can restrict the entry of a broad range of enveloped and nonenveloped viruses (Brass et al., 2009; Huang et al., 2011; Anafu et al., 2013; Bailey et al., 2014). Mudhasani et al. demonstrated that depletion of IFITM2 and IFITM3 but not IFITM1 resulted in enhanced RVFV infection, indicating that IFITM2 and IFITM3 are antiviral against RVFV (Mudhasani et al., 2013). The authors performed a series of experiments and found that IFITM2 and IFITM3 blocked RVFV membrane fusion with the endosomes but did not affect viral receptor binding, internalization or replication. They also extended the antiviral activity of IFITMs into other bunyaviruses, including LACV, HTNV, and ANDV. In contrast to RVFV, IFITM1 was found to block LACV, HTNV and ANDV to a comparable level as IFITM2 and IFITM3. Most importantly, a high frequency of single nucleotide polymorphism (SNP) rs12252 of IFITM3 with impaired antiviral function against HTNV was found in severe hemorrhagic fever with renal syndrome (HFRS) patients. These data highlight a critical role of IFITM3 in disease severity in HFRS (Xu-Yang et al., 2017). Interestingly, CCHFV and several strains of arenaviruses were resistant to IFITMs restriction (Mudhasani et al., 2013; Stott-Marshall and Foster, 2022). Future studies are needed to determine the specificity of IFITMs against different bunyaviruses.

IFN-stimulated gene 20 (ISG20): ISG20 is a member of 3′ to 5′ exonuclease superfamily that has been shown to suppress the replication of diverse RNA and DNA viruses (Espert et al., 2003; Zhou et al., 2011; Liu et al., 2017). Using gain-of-function genetic screening with lentiviral vectors encoding 488 ISGs from humans and rhesus macaques, Feng et al. identified ISG20 as a restriction factor that blocked BUNV replication (Feng et al., 2018). To explore the spectrum of ISG20 antiviral activity, the authors screened a panel of bunyaviruses and observed that ISG20 inhibited most viruses tested, but had no effect on phleboviruses, including RVFV, HRTV and SFTSV. Depletion of endogenous ISG20 led to increased viral infection in the absence of IFN treatment, suggesting that the basal level of ISG20 was sufficient to restrict BUNV. ISG20 was shown to block viral replication by degrading viral genes (Espert et al., 2003). Indeed, the gene expression of all three BUNV segments was inhibited by ISG20. The antibunyaviral activity of ISG20 depended on its exonuclease function because the catalytically defective mutants abolished their antiviral effect. Although multiple mutations have been identified in the genome of BUNV that was resistant to ISG20, the mechanisms by which BUNV counteracts ISG20 restriction remain to be elucidated (Feng et al., 2018).

Moloney leukemia virus 10 protein (MOV10): MOV10 was originally identified as a host protein that blocked Moloney murine leukemia virus infection in mice (Mooslehner et al., 1991). MOV10 is a 5’ to 3’ RNA helicase that is involved in many cellular processes related to RNA metabolism (Nawaz et al., 2022). MOV10 was also found to play a role in innate immune response against viral infection via modulation of IFN signaling (Cuevas et al., 2016; Yang et al., 2022). Using affinity purification coupled with mass spectrometry, MOV10 was identified as an interactor of SFTSV N protein in an RNA-independent manner (Mo et al., 2020). In addition to SFTSV, MOV10 was shown to associate with the N proteins of two related bunyaviruses: HRTV and GTV. Depletion of MOV10 promoted viral infection of SFTSV, HRTV and GTV, suggesting an antiviral role of MOV10 against these viruses. Interestingly, the antiviral function of MOV10 was independent of its helicase activity and IFN signaling. Rather, MOV10 interacted with N-arm domain of SFTSV N protein via its N-terminus and blocked N protein polymerization and the interactions of N protein and viral RNA as well as viral polymerase, therefore disrupting the assembly of viral ribonucleoprotein (RNP). These data suggest that MOV10 functions as a restriction factor against bunyaviruses tested. However, given the low sequence identity of N protein found in bunyaviruses, it is currently unknown whether and how MOV10 restricts the infection of other bunyaviruses (Mo et al., 2020).

Myxovirus resistance protein A (MxA): Human MxA and MxB (known as Mx1 and Mx2, respectively in rodent) belong to the dynamin-like large guanosine triphosphatases (GTPases) that are involved in intracellular vesicle trafficking (Haller et al., 2007). Mouse Mx1 has been originally linked to conferring resistance to IAV in a IFN-dependent manner (Haller et al., 1979). Later studies revealed that Mx proteins from humans and rodents have broad antiviral activities against a wide range of viruses, including bunyaviruses (Haller et al., 2015). Ectopic expression of MxA blocked LACV and RVFV infection at an early stage of viral life cycle (Frese et al., 1996). Mechanistically, MxA was demonstrated to interact with LACV N protein at COP-I positive vesicular-tubular membranes of ER-Golgi intermediate compartment via its C-terminal domain, leading to the sequestration of N protein that is required for viral genome replication (Kochs et al., 2002; Reichelt et al., 2004). Both GTP binding and hydrolysis in N-terminal domain were found to be required for MxA relocalization to target bunyavirus N protein and block viral infection (Dick et al., 2015; Mckellar et al., 2023). Furthermore, MxA was shown to block the primary transcription of RVFV, therefore inhibiting viral RNA synthesis (Habjan et al., 2009a). In addition to LACV N protein, a recent study showed that MxA interacted with SFTSV N protein to disrupt the interaction of N protein and viral polymerase and suppress RNP activity (Chang et al., 2024). The MxA-mediated restriction was also extended to other bunyaviruses including Puumala and Tula hantaviruses and other species (Kanerva et al., 1996). Similar to human MxA, bat MxA was shown to impair the activity of RVFV polymerase using a minigenome system (Fuchs et al., 2017). Interestingly, rat Mx2, but not rat Mx1 blocked LACV and RVFV (Sandrock et al., 2001; Stertz et al., 2007). Importantly, MxA restricted LACV infection in the MxA-transgenic mice without IFN receptors, indicating a role of MxA in blocking viral infection *in vivo* (Hefti et al., 1999). Together, these data suggest an evolutionarily conserved role of Mx proteins in host defense against bunyaviruses.

Protein kinase R (PKR): PKR is an IFN-induced dsRNA-dependent serine/threonine-protein kinase that is involved in host innate immunity against viral infection (Dauber and Wolff, 2009). Upon viral infection, PKR can be activated by viral dsRNA, leading to the phosphorylation of the alpha subunit of eukaryotic translation initiation factor 2 (eIF2α) to block the synthesis of both cellular and viral proteins (García et al., 2006). While both wild-type (WT) and NSs-deleted (ΔNSs) BUNV were able to trigger PKR activation and induce eIF2α phosphorylation to a similar level, the expression of PKR in HEK293 cells inhibited WT BUNV but had moderate effect on ΔNSs BUNV (Streitenfeld et al., 2003; Carlton-Smith and Elliott, 2012). In contrast, loss of PKR potently increased the infection of ΔNSs RVFV but not WT RVFV, suggesting that NSs protein of RVFV may antagonize PKR restriction. Indeed, RVFV NSs was shown to promote the degradation of PKR in a proteasome-dependent manner, therefore suppressing eIF2α phosphorylation (Ikegami et al., 2009; Habjan et al., 2009b). The NSs-mediated downregulation of PKR appears to be in a virus-dependent manner, because LACV NSs had no effect on PKR expression (Habjan et al., 2009b). Importantly, PKR knockout mice were more susceptible to BUNV and RVFV than WT mice, suggesting that PKR is antiviral against BUNV and RVFV *in vivo* (Streitenfeld et al., 2003; Habjan et al., 2009b). In addition to NSs-induced downregulation of PKR, bunyaviruses employ other strategies to counteract PKR restriction and inhibit PKR-dependent stress granule formation (Christ et al., 2020). For example, ANDV N protein was found to inhibit PKR dimerization and autophosphorylation (Wang and Mir, 2015). Mechanistically, ANDV N protein interacted with P58^IPK^, an endogenous PKR inhibitor, and recruited P58^IPK^ to the 40S ribosomal subunit to suppress PKR activation (Wang et al., 2021).

Virus inhibitory protein, endoplasmic reticulum-associated, interferon-inducible (Viperin): Viperin is a radical S-adenosyl-l-methionine (SAM) domain-containing enzyme that has been implicated in the restriction of a broad range of RNA and DNA viruses (Shen et al., 2014; Van Der Hoek et al., 2017; Ghosh and Marsh, 2020). Using HEK293 cells expressing individual ISGs, Viperin was identified as an ISG that potently blocked both WT and ΔNSs BUNV infection (Carlton-Smith and Elliott, 2012). The basal expression level of Viperin in most cell types is low but it can be induced by IFN treatment or viral infection (Helbig et al., 2013). Interestingly, ΔNSs BUNV but not WT BUNV was able to induce Viperin expression, suggesting a role of NSs in counteracting IFN signaling to induce antiviral ISGs. Viperin has been reported to impact many stages of viral life cycle, including transcription, replication, assembly and release (Wang et al., 2007; Helbig et al., 2011; Bernheim et al., 2021). In addition, Viperin’s enzymatic product 3′-deoxy-3′,4′-didehydro-CTP (ddhCTP) is also involved in Viperin-mediated restriction by functioning as a chain terminator for RNA-dependent RNA polymerase of flaviviruses (Gizzi et al., 2018). Although the enzymatic function of Viperin was found to be required to inhibit BUNV infection, the precise mechanism of its antiviral activity remains elusive (Carlton-Smith and Elliott, 2012).

## Antagonism of type I IFN response by bunyavirus

**Figure 2:**
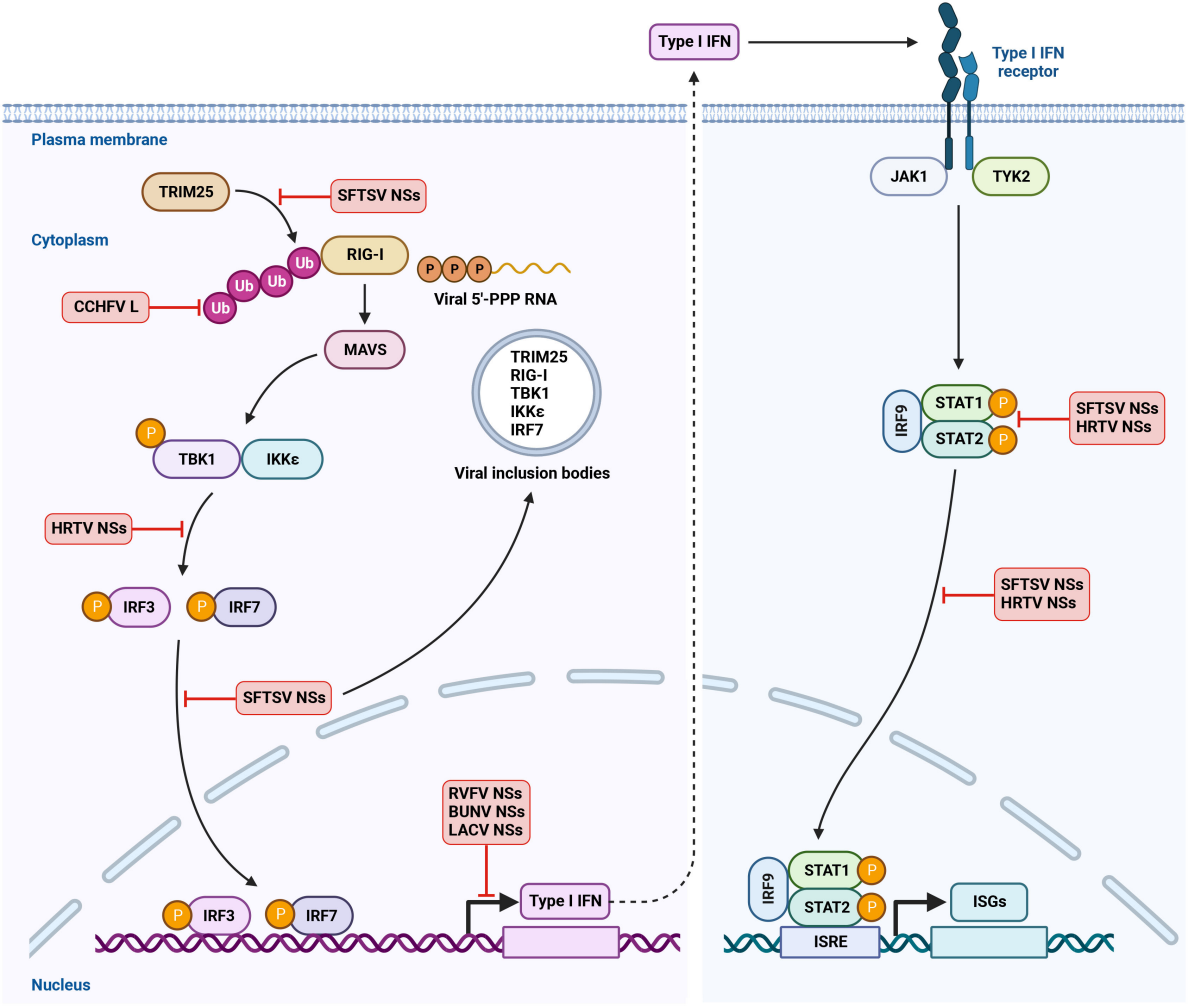
Antagonism of IFN induction and IFNAR signaling by bunyavirus. RIG-I is the major immune sensor to detect bunyavirus infection by recognition of viral 5’-PPP RNA and other viral RNA species in the cytoplasm. Upon binding to viral RNA ligands, RIG-I multimerizes and interacts with adaptor protein MAVS, leading to the activation of TBK1 and IKKϵ. TRIM25-mediated Lys 63-linked ubiquitination is critical for RIG-I activation. TBK1 and IKKϵ then phosphorylate the transcription factors IRF3 and IRF7. The phosphorylated IRF3 and IRF7 translocate into nucleus and activate IFN transcription to produce IFN. Type I IFN binds to IFN receptor IFNAR on the cell surface and triggers the phosphorylation of kinases JAK1 and TYK2. JAK1 and TYK2 then phosphorylate the transcription factors STAT1 and STAT2. The activated STAT1 and STAT2 recruit IRF9 and translocate into nucleus to induce the transcription of hundreds of ISGs. This confers an antiviral state to the host cells. Bunyaviruses have evolved multiple mechanisms to antagonize IFN production and IFNAR signaling. Created with BioRender.com.

To overcome IFN-meditated restriction and spread in the host cells, bunyaviruses have evolved effective strategies to block IFN production or downstream IFN signaling. Two attenuated RVFV strains MP12 and clone 13 that contain mutations in NSs were shown to strongly induce the expression of IFN-α and IFN-β and were virulent only in IFN-α/IFN-β receptor knockout mice. In contrast, RVFV virulent strain ZH548 was unable to stimulate IFNs and efficiently replicated in both IFN-competent and IFN-deficient mice. This study provided the first hint that RVFV NSs may be an IFN antagonist and contribute to RVFV virulence and pathogenesis (Bouloy et al., 2001). Indeed, WT RVFV but not RVFV lacking NSs inhibited the expression of IFN-α2, IFN-β and TNF-α, confirming the role of RVFV NSs in antagonizing host immune response (Mcelroy and Nichol, 2012). In addition to RVFV NSs, the NSs proteins of other bunyaviruses including BUNV, LACV, OROV, HRTV and SFTSV have been shown to antagonize IFN pathways (Bridgen et al., 2001; Blakqori et al., 2007; Qu et al., 2012; Tilston-Lunel et al., 2016; Taniguchi et al., 2022). Here, we discuss the molecular strategies employed by bunyaviruses to counteract type I IFN response (**Figure 2**).

### Inhibition of host cellular transcription

Bunyavirus NSs-mediated inhibition of IFN production has been linked to its ability to suppress the transcription of host cellular RNA. Indeed, RVFV NSs was found to interact with p44 subunit of host transcription factor TFIIH and cause the sequestration of p44 and XPB subunits of TFIIH in the nuclear filament to disrupt the assembly of TFIIH, leading to the inhibition of host RNA synthesis (Le May et al., 2004). In addition to p44 and XPB subunits, RVFV NSs associated with p62 subunit of TFIIH via its ΩXaV motif and promoted the proteasomal degradation of p62 by interacting with E3 ubiquitin ligase FBXO3 (Kalveram et al., 2011; Kainulainen et al., 2014; Cyr et al., 2015). Moreover, RVFV NSs specifically blocked IFN-β gene expression in the absence of interfering with other IFN transcription factors including IRF3 and NF-κB (Billecocq et al., 2004). Mechanically, RVFV NSs interacted with SAP30, a member of Sin3A/NCoR/HDACs repressor complexes and associated with transcription factor YY1 that is involved in the regulation of IFN-β gene expression. In RVFV infected cells, NSs, SAP30 and other repressor proteins were recruited into IFN-β promoter via YY1 and suppressed IFN-β transcription (Le May et al., 2008).

Similar to RVFV, BUNV NSs protein was found to potently antagonize IFN induction (Bridgen et al., 2001; Weber et al., 2002). BUNV NSs was shown to either directly inhibit RNA polymerase II phosphorylation or indirectly disrupt RNA polymerase II function by interacting with MED8, a host protein that regulates RNA polymerase II-mediated transcription, resulting in the suppression of host gene expression (Thomas et al., 2004; Léonard et al., 2006; Van Knippenberg et al., 2010). Moreover, LACV NSs interfered with RNA polymerase II by promoting the proteasomal degradation of its RPB1 subunit through E3 ubiquitin ligase Elongin C (Blakqori et al., 2007; Verbruggen et al., 2011; Schoen et al., 2020).

### Suppression of RIG-I-mediated sensing pathway

RIG-I is the major immune sensor to detect bunyavirus infection. In response, bunyavirus can target and interfere with RIG-I or other downstream signaling molecules to block type I IFN induction. Indeed, HRTV NSs has been shown to associate with TBK1 and disrupt TBK1-IRF3 interaction, leading to the suppression of IRF3 activation and IFN production (Ning et al., 2017). Moreover, SFTSV NSs was found to interact with several key components of RIG-I-mediated sensing pathway including RIG-I, TBK1, IKKϵ, IRF7 and TRIM25, an E3 ubiquitin ligase that is required for RIG-I activation, and cause the sequestration of these proteins from cytoplasm into viral inclusion bodies, therefore inhibiting IFN signaling transduction (Qu et al., 2012; Ning et al., 2014; Santiago et al., 2014; Hong et al., 2019; Min et al., 2020). In line with these studies, NSs mutant that was deficient for the formation of viral inclusion bodies lost its ability to restrict IFN production, highlighting a role of SFTSV NSs-induced inclusion bodies in the antagonism of IFN response (Ning et al., 2014). SFTSV NSs was also shown to block the phosphorylation and dimerization of IRF3 via interaction with LSm14A, a host protein involved in IRF3 activation (Zhang et al., 2021). In addition to NSs protein, other bunyaviral proteins can counteract innate immunity. For example, HTNV Gn protein was reported to induce mitophagy to promote MAVS degradation, leading to the inhibition of type I IFN response (Wang et al., 2019). Furthermore, ectopic expression of SFTSV N protein was shown to inhibit IFN-β promoter activation (Qu et al., 2012). However, the inhibitory effect of SFTSV N on IFN production in the context of viral infection needs to be further investigated.

Many proteins in host innate immune sensing pathways are regulated by post-translational modifications including ubiquitination and ISG15-mediated ISGylation. Ubiquitination is required for the activation of multiple key signaling molecules such as RIG-I, MAVS, TBK1, and IRF3 (Hu and Sun, 2016). Cellular ubiquitination is highly regulated by deubiquitinases (DUBs) via removing ubiquitin from target proteins. DUBs are classified into five families and one of these families is called Ovarian tumor proteases (OTU) (Komander et al., 2009). Interestingly, in addition to eukaryotic proteins, OTU was found in several viral proteins, suggesting that virus may utilize its DUB to interfere with host ubiquitination and disrupt RIG-I-dependent IFN signaling. Indeed, CCHFV L protein was shown to contain an OTU domain that encodes a cysteine protease with DUB activity (Makarova et al., 2000; Bailey-Elkin et al., 2014). Overexpression of CCHFV L protein decreased the ubiquitination and ISGylation of cellular proteins and attenuated both ISG15- and NF-κB-mediated antiviral response (Frias-Staheli et al., 2007). Furthermore, using reverse genetics, CCHFV variant that was deficient for DUB activity was shown to promote immune response, further confirming the role of CCHFV OTU as an IFN antagonist in the context of viral infection (Scholte et al., 2017). Intriguingly, a synthetic ubiquitin variant with high-affinity binding to CCHFV OTU domain was found to not only relieve OTU-mediated immune antagonism but also inhibit viral infection by blocking viral RNA synthesis, demonstrating that CCHFV OTU can function as a therapeutic target to control viral infection (Scholte et al., 2019).

### Antagonism of IFNAR signaling

IFNs bind to their receptors on the cell surface and activate JAK/STAT signaling pathway, leading to the transcription of hundreds of ISGs to block viral infection. Bunyaviruses have developed mechanisms to overcome IFNAR signaling to prevent the induction of antiviral ISGs. SFTSV NSs has been shown to interact with STAT2 and relocalize STAT1 and STAT2 into viral inclusion bodies to block the phosphorylation and subsequent nuclear translocation of STAT proteins, resulting in the suppression of ISG expression (Ning et al., 2015; Chen et al., 2017). In addition to sequestering STAT proteins and dampening their phosphorylation, SFTSV infection led to the downregulation of STAT1. However, the mechanisms by which SFTSV decreases STAT1 protein abundance remain elusive (Ning et al., 2019). Similar to SFTSV NSs, HRTV NSs interacted with STAT2 and impaired the phosphorylation and nuclear translocation of STAT2, suggesting a conserved function of bunyavirus NSs proteins in antagonizing IFNAR signaling (Feng et al., 2019).

## Concluding remarks

IFN-mediated innate immunity is critical for the control of bunyavirus infection and has been linked to viral pathogenesis and disease progression in patients infected with bunyaviruses. Indeed, the serum IFN-β level of SFTS patients was negatively correlated with serum viral load and the dysregulated IFN responses including impaired expression of IFN-β and TLR3 were observed in fatal SFTS patients (Song et al., 2017). Additionally, the polymorphisms of human genes involved in the innate immune system, including RIG-I, MAVS and several TLRs were found to be associated with critical illness in RVFV infected individuals (Hise et al., 2015).

As a major response to IFN signaling, hundreds of ISGs are produced. Although several ISGs have been shown to have antiviral activities against bunyaviruses in cell culture-based assays, their roles in the restriction of viral infection *in vivo* and viral pathogenesis need to be further determined by using the relevant animal models. Furthermore, how human polymorphisms of these ISGs contribute to disease severity caused by bunyavirus infection is largely unknown. Another challenge is how to translate our knowledge of these antiviral ISGs into the strategies to design and develop antiviral agents. One approach is to screen small drug-like molecules that can induce certain ISGs at the transcriptional level to block viral infection in the absence of global IFN induction. This method could avoid the potential side effects caused by IFN treatment clinically.

Although bunyaviruses are highly diverse genetically, they have evolved to employ common strategies to antagonize IFN response. NSs protein of bunyavirus is one of such IFN antagonists and has been extensively investigated. Virus lacking NSs was found to be a strong IFN inducer and has less virulent compared to WT strain, therefore having the potential for a vaccine candidate. Indeed, RVFV clone 13 that has large natural deletion in the NSs gene was shown to be efficacious and safe as a live-attenuated candidate vaccine for different species such as sheep, goats, and cattle (Dungu et al., 2010). The efforts that have been made to improve the efficacy and safety of the vaccine candidates by deleting virulence factors including NSs gene using reverse genetics will eventually contribute to the development of human vaccines against RVFV and other bunyaviruses. A more detailed understanding of host innate immunity-mediated restriction of virus and the mechanisms of viral immune evasion will not only further our knowledge of virus pathogenesis, but also facilitate the development of antivirals and vaccines to combat viral infection.

## Author contributions

ML: Writing – review & editing, Writing – original draft.
